# Supplementary material for: PTEN is a protein phosphatase that targets active PTK6 and inhibits PTK6 oncogenic signaling in prostate cancer
Source: Nat Commun. 2017 Nov 15;8:1508. doi: 10.1038/s41467-017-01574-5 (PMC5688148; doi:10.1038/s41467-017-01574-5)

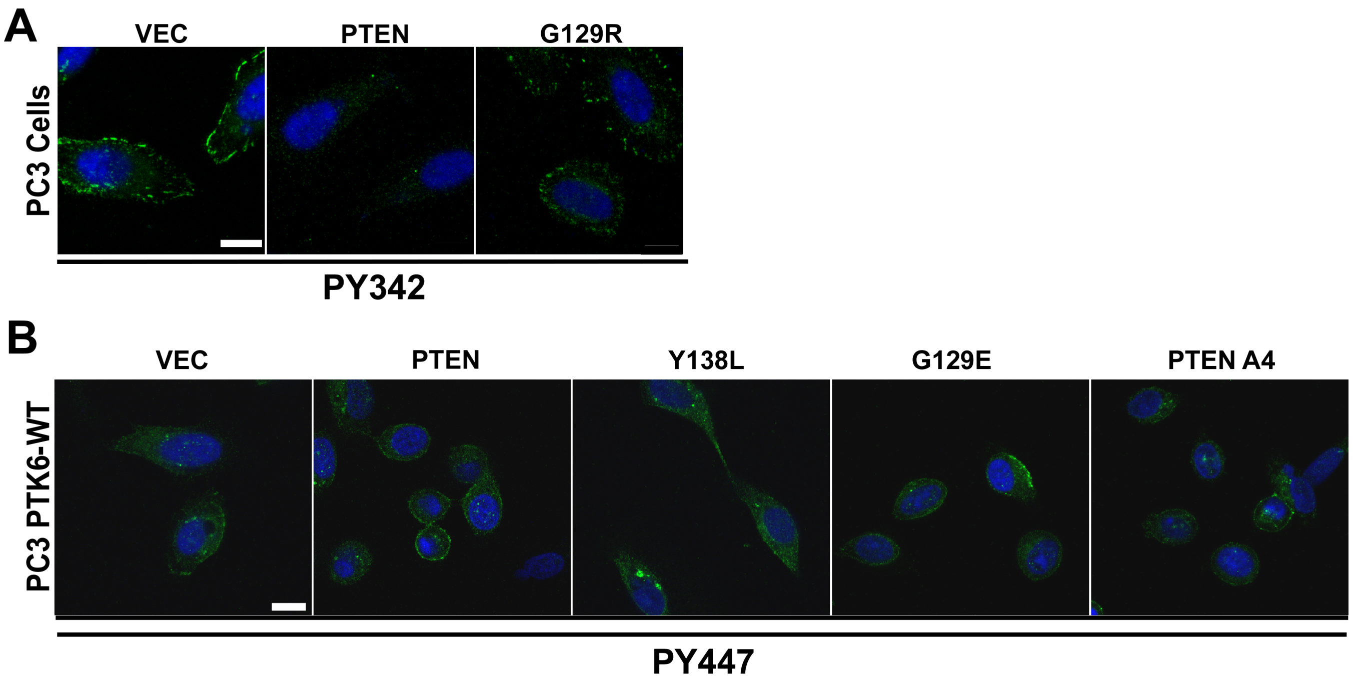

Supplementary Figure 1. Localization of active (PY342) and inactive (PY447) PTK6 in PC3 cells expressing different PTEN constructs. (A) Expression of wild type PTEN targets PY342 at the plasma membrane. PC3 cells stably expressing empty vector (VEC), PTEN, or PTEN G129R (catalytically inactive) were stained for endogenous active PTK6 (PY342, green). Cells were counterstained with DAPI (blue). Scale bar, 10 μm. (B) PC3 cells stably expressing PTK6-WT were transiently transfected with empty vector, wild-type PTEN, or PTEN mutants G129E, Y138L, or PTEN A4. PTK6 PY447 was visualized by immunofluorescence (green), and was not targeted by PTEN. The corresponding immunoblots are shown in Figure 4B. Cells were counterstained with DAPI (blue). Scale bar, 10 μm.

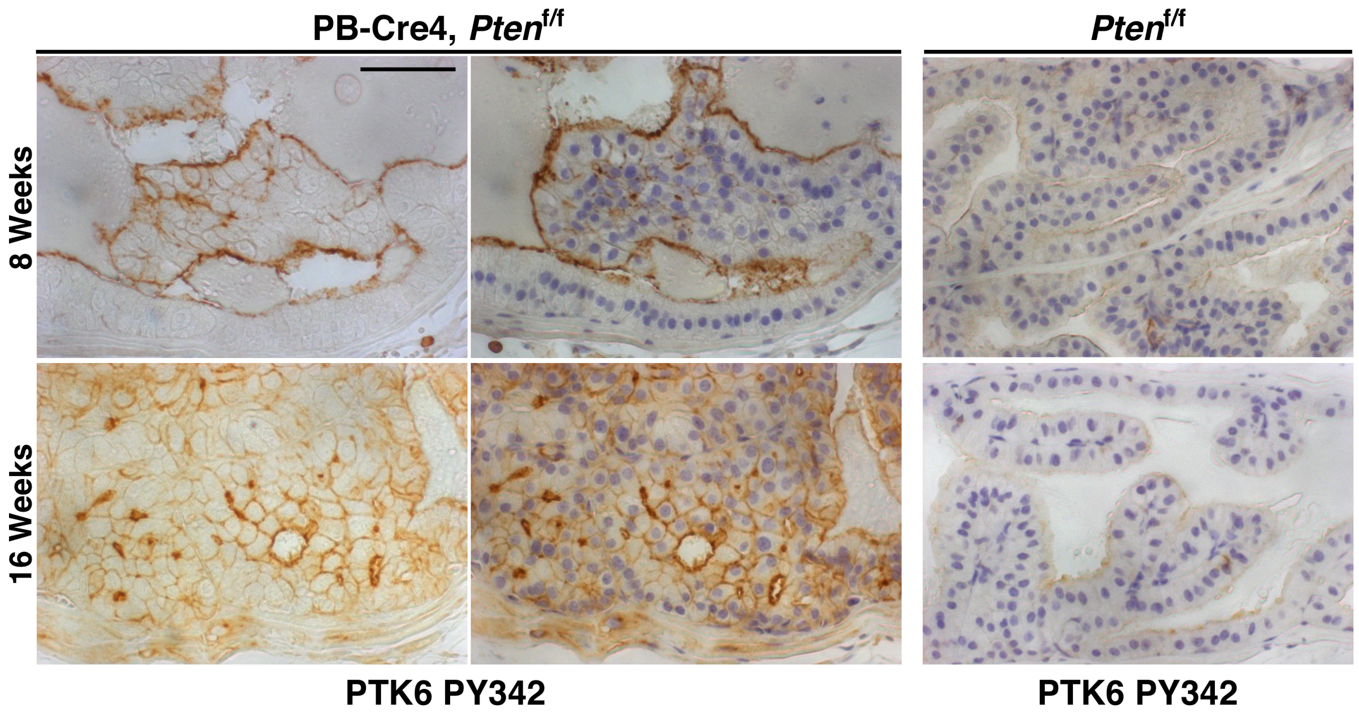

Supplementary Figure 2. Activation of PTK6 (PY342) was examined in *Pten* wild type (*Pten*$^{f/f}$) and *Pten*-deficient (PB-Cre4, *Pten*$^{f/f}$) prostates at 8 and 16 weeks of age using immunohistochemistry. Unstained and hematoxylin counterstained sections are shown, with immunoreactivity detected using DAB (brown). At eight weeks of age activation of PTK6 is most striking at the apical membrane in PB-Cre4, Pten$^{f/f}$ prostates. At 16 weeks basolateral localization of PY342 was evident in PB-Cre4, Pten$^{f/f}$ prostates. Active PTK6 was not detected in control (*Pten*$^{f/f}$) prostates. Scale bar, 50 μm.

Additional supplementary files: Scans of uncropped films for Figures 1 - 4.

Figure 1-A

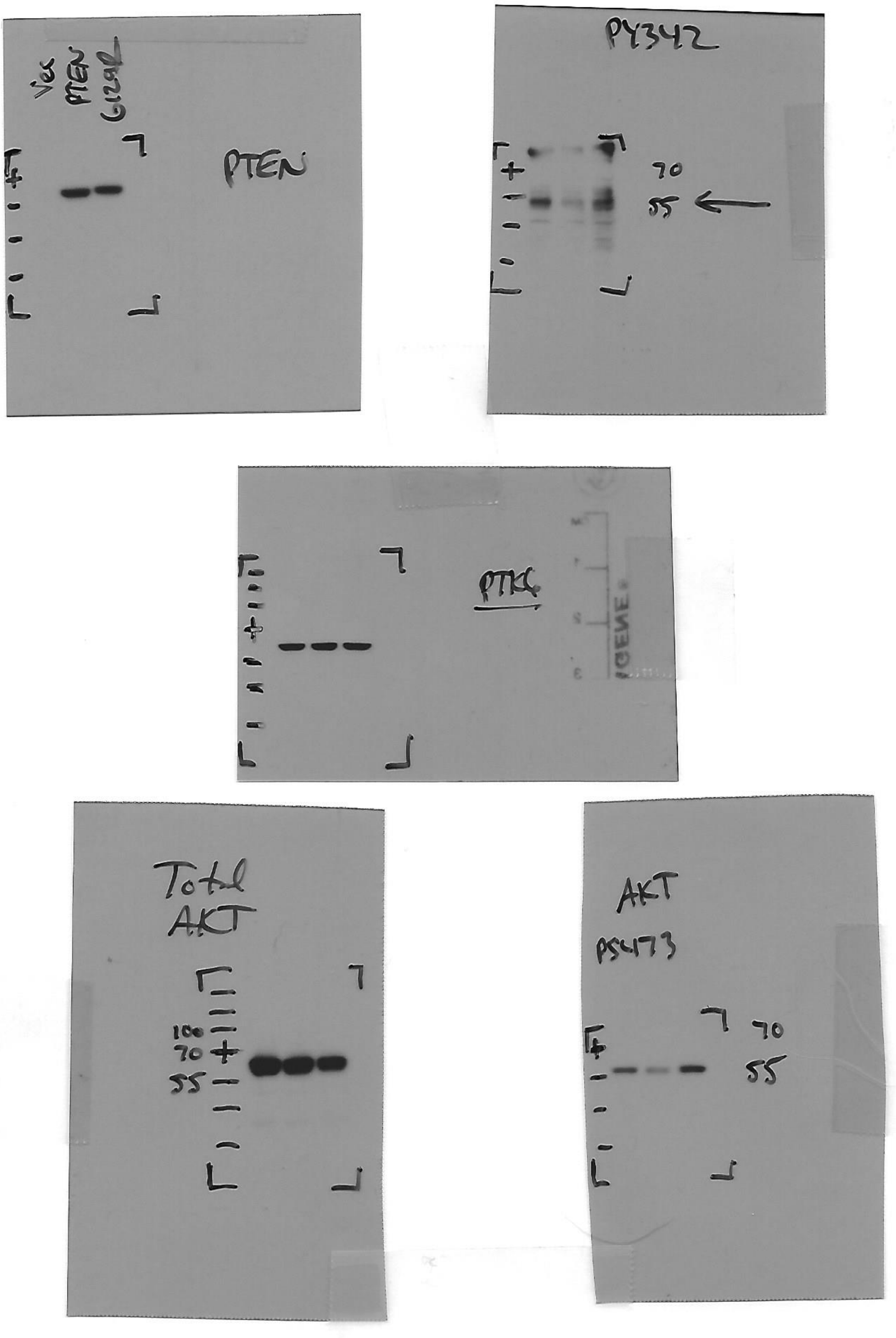

Vec
PTEN
G129E
PTEN
PY342
70
55
PTK6
Total
AKT
100
70
55
AKT
70
55

Figure 1 - B

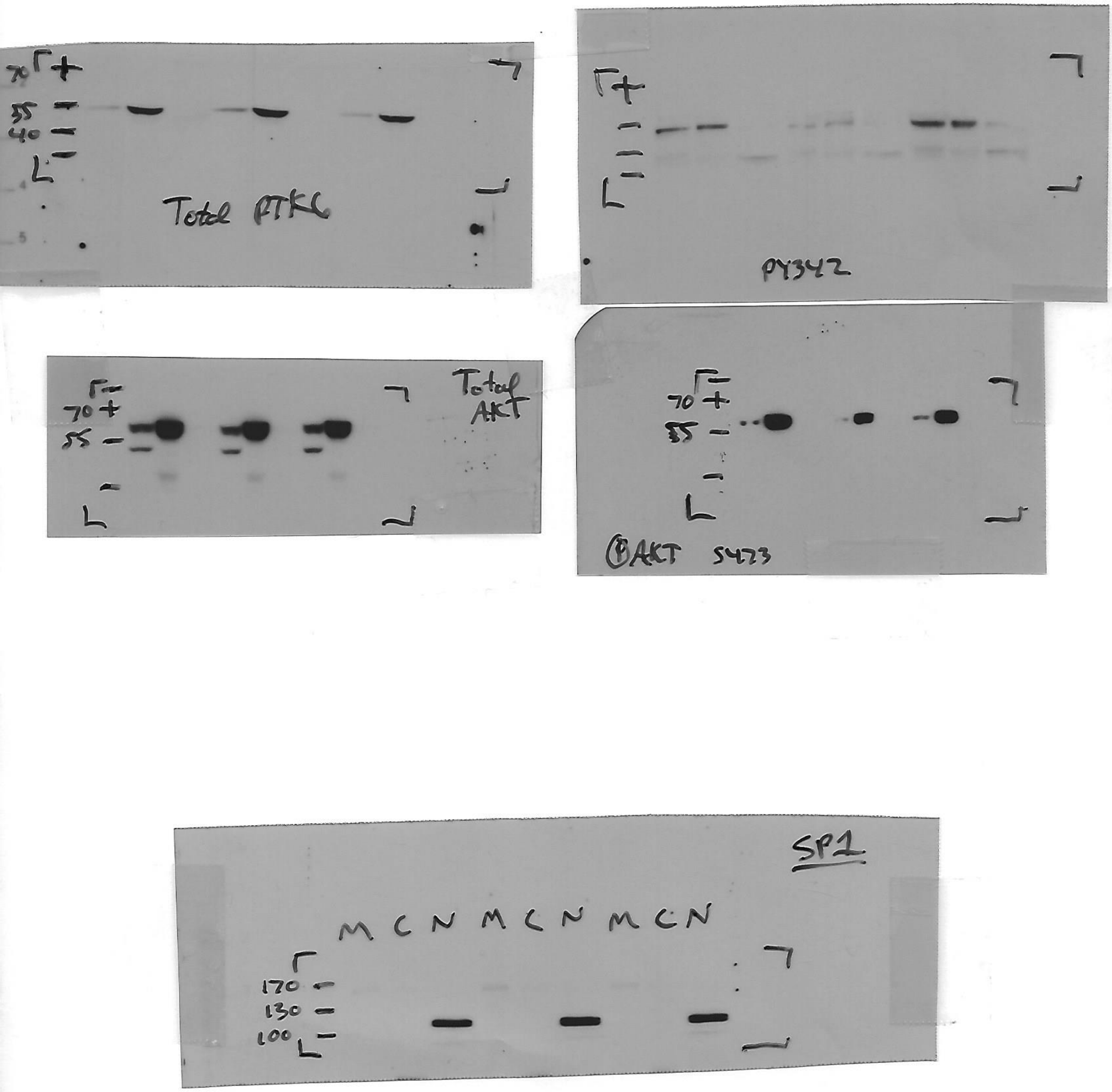

55
40
Total PTK6
PY342
70
55
Total AKT
70
55
AKT
SP1
M C N M C N M C N
170
130
100

Figure 1- C

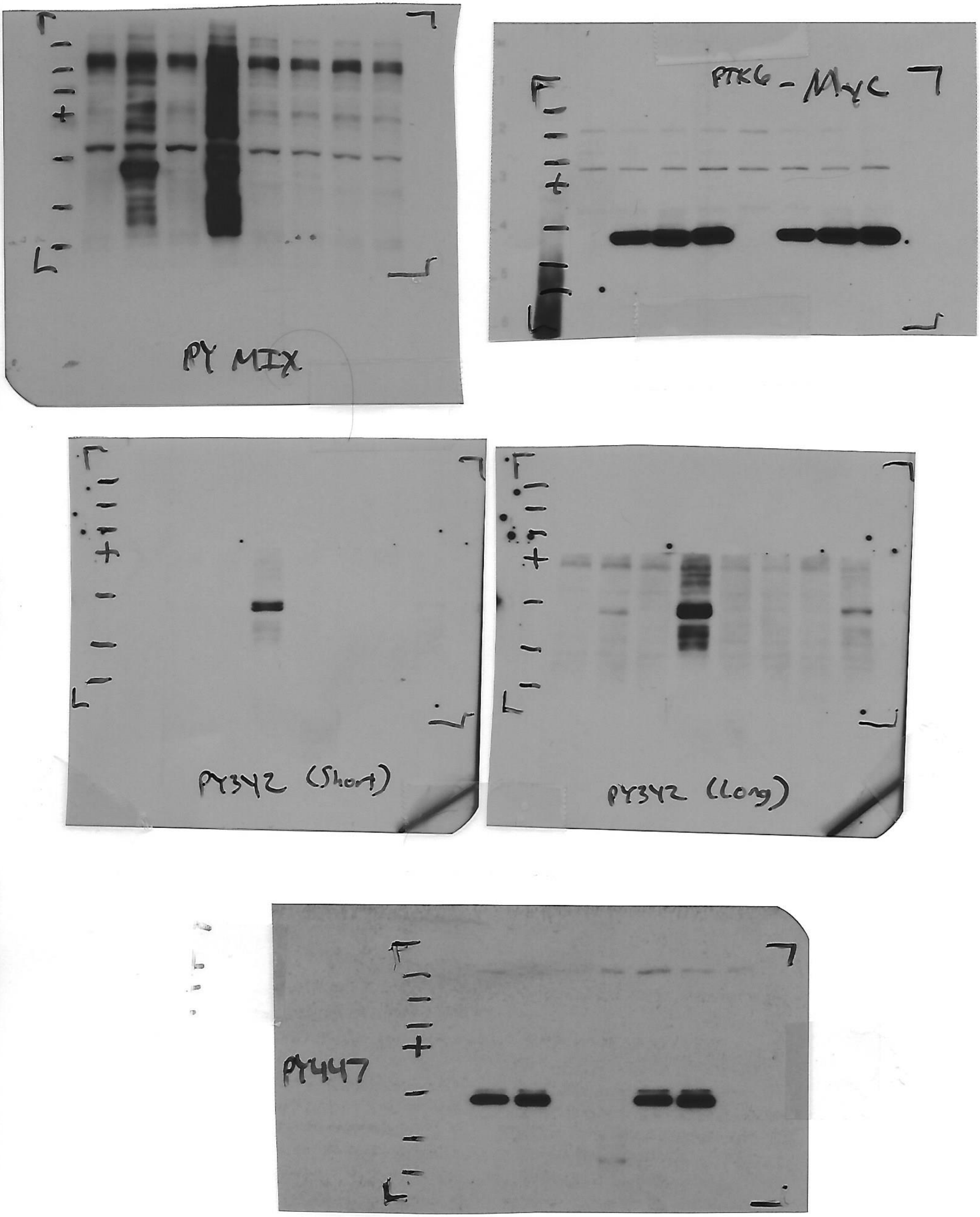

Figure 1-C

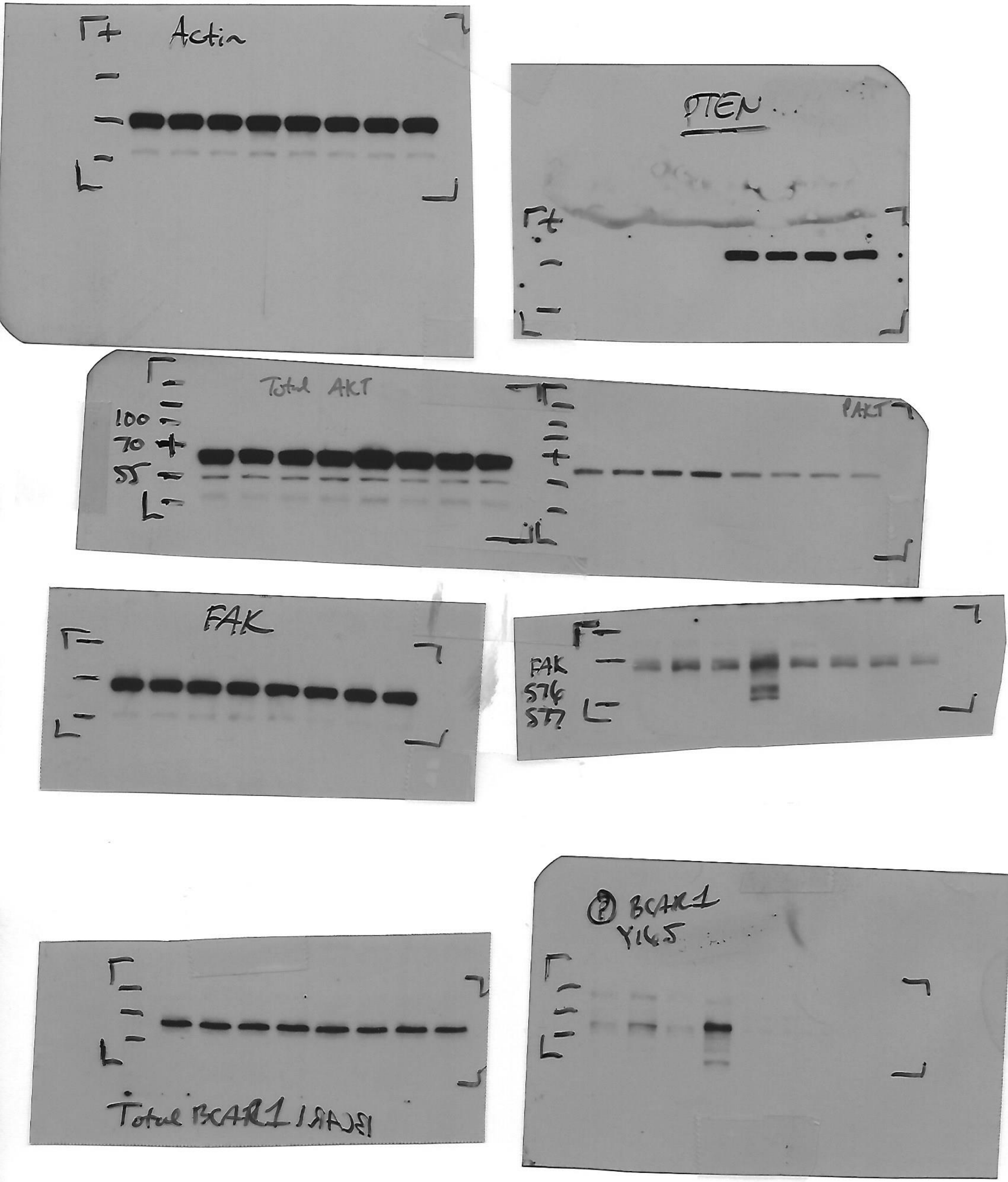
Actin
PTEN
Total AKT
100
70
55
pAKT
FAK
FAK
576
577
BCAR1
Y165
Total BCAR1

Figure 1-E

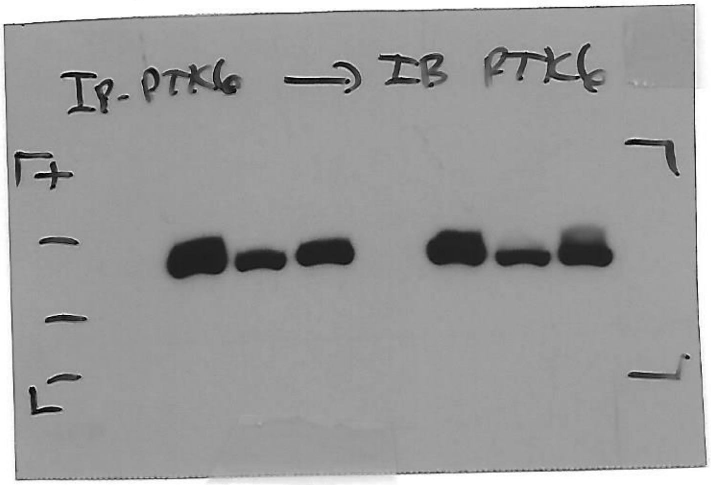
IP-PTK6 → IB PTK6

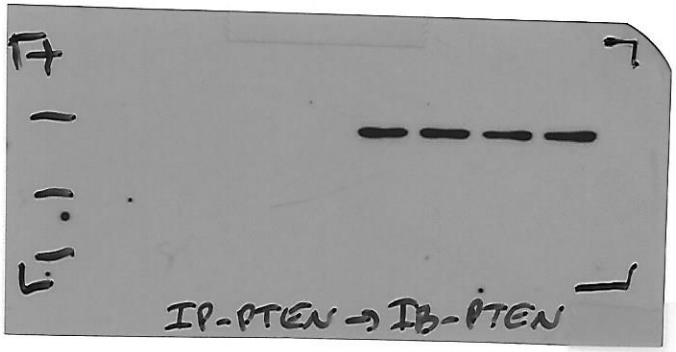
IP-PTEN → IB-PTEN

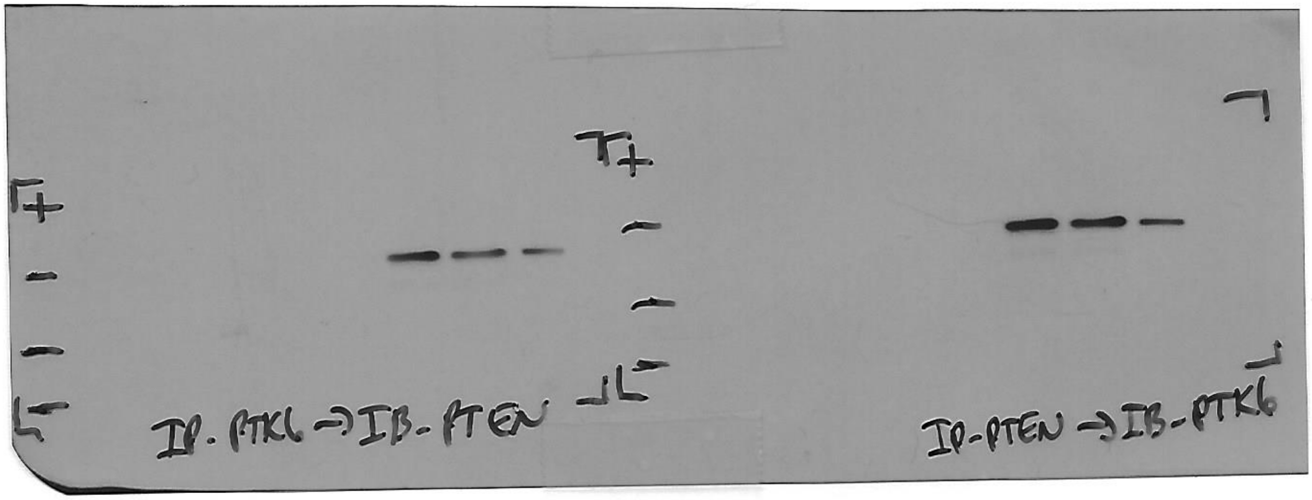
IP-PTK6 → IB-PTEN
IP-PTEN → IB-PTK6

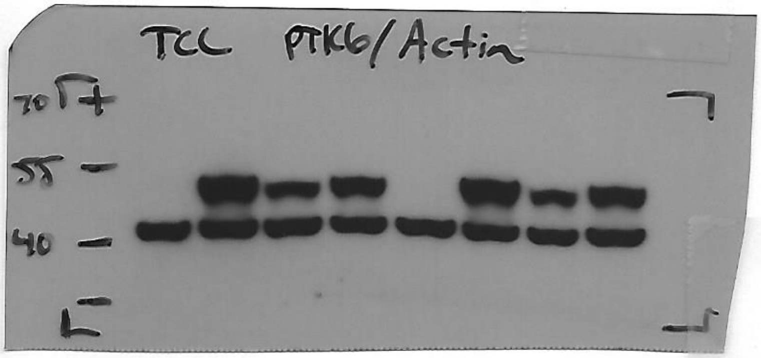
TCL PTK6/Actin
70
55
40

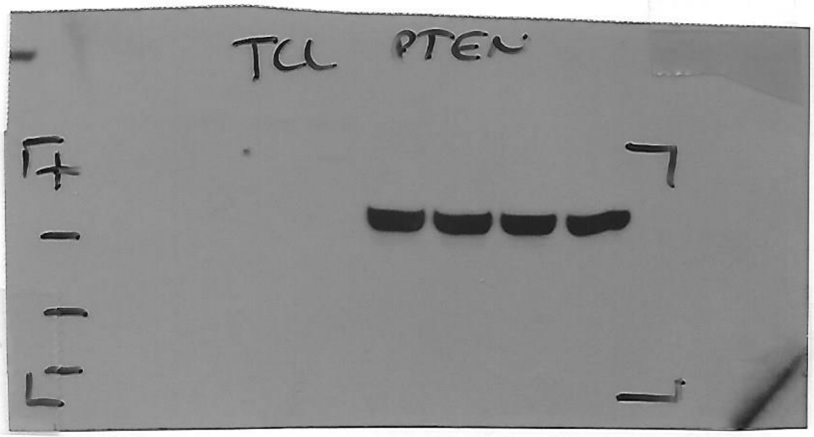
TCL PTEN

Figure 1F

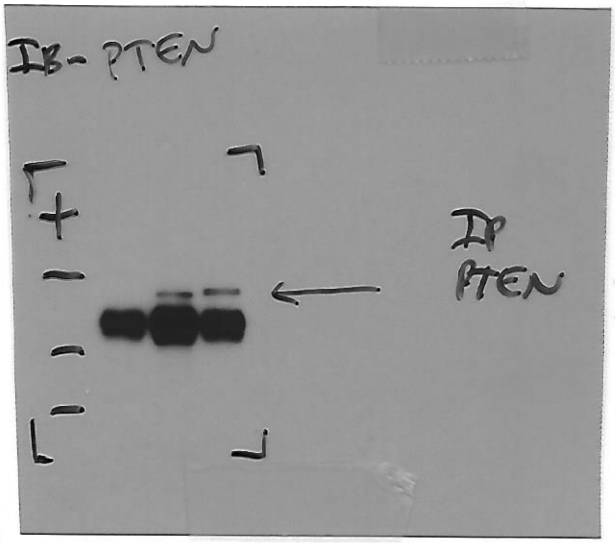
IB- PTEN
IP
PTEN

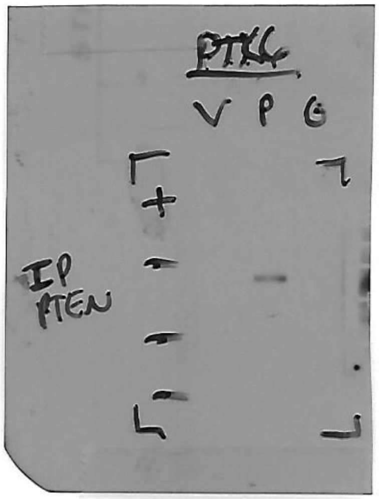
PTK6
V P G
IP
PTEN

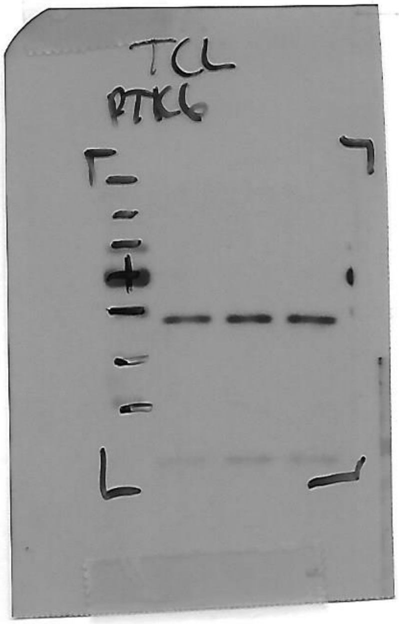
TCL
PTK6

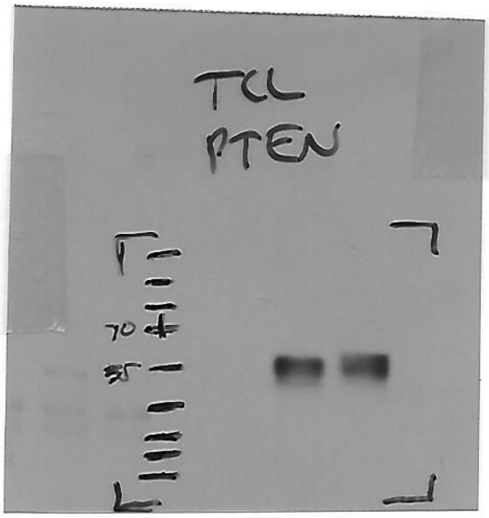
TCL
PTEN
70
55

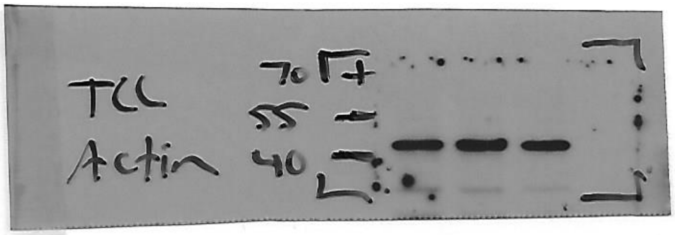
TCL
Actin
70
55
40

Figure 2 -A

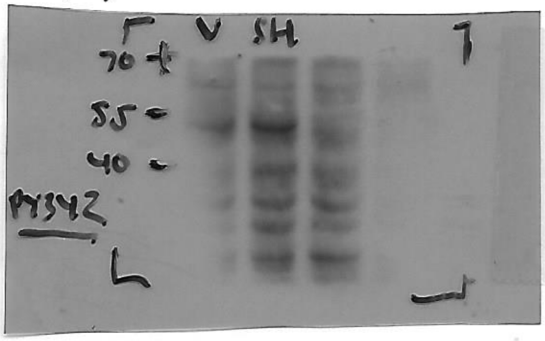
V SH
70
55
40
PY342

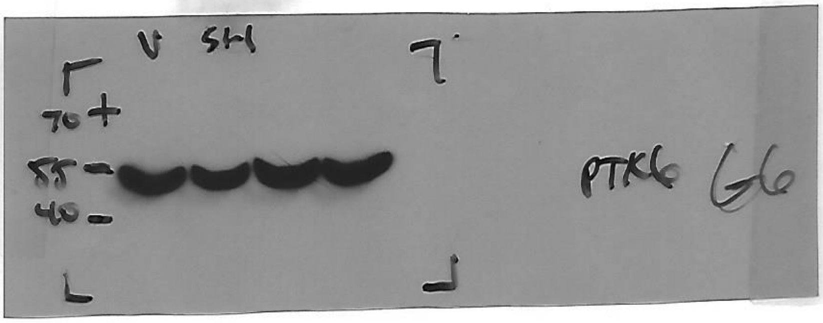
V SH
70
55
40
PTK6
G6

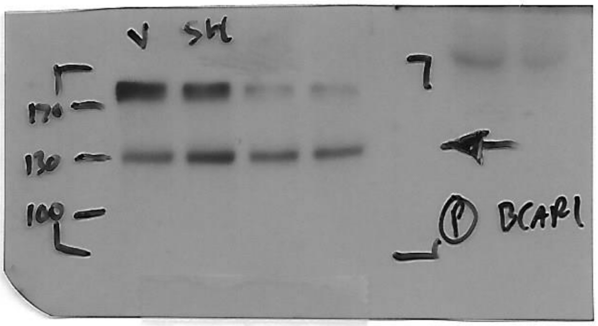
V SH
170
130
100
Ⓟ BCAR1

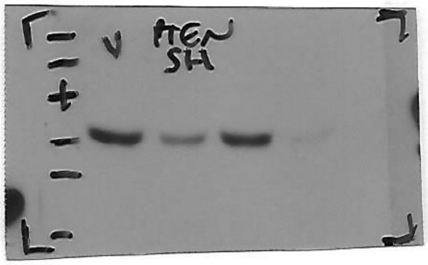
V PTEN SH

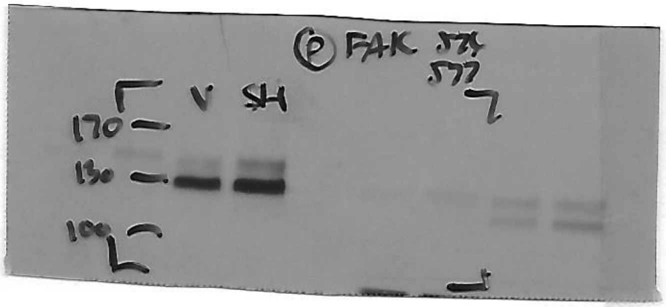
Ⓟ FAK 575 577
V SH
170
130
100

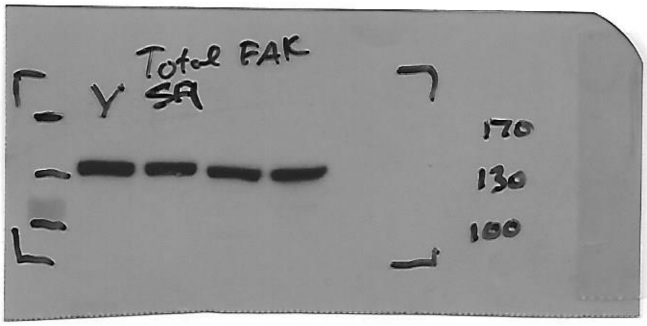
Total FAK
V SH
170
130
100

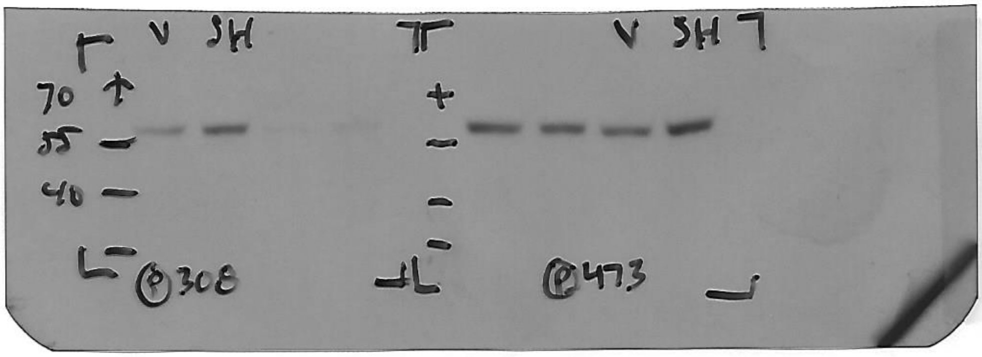
V SH
V SH
70
55
40
Ⓟ308
Ⓟ473

Figure 2-A

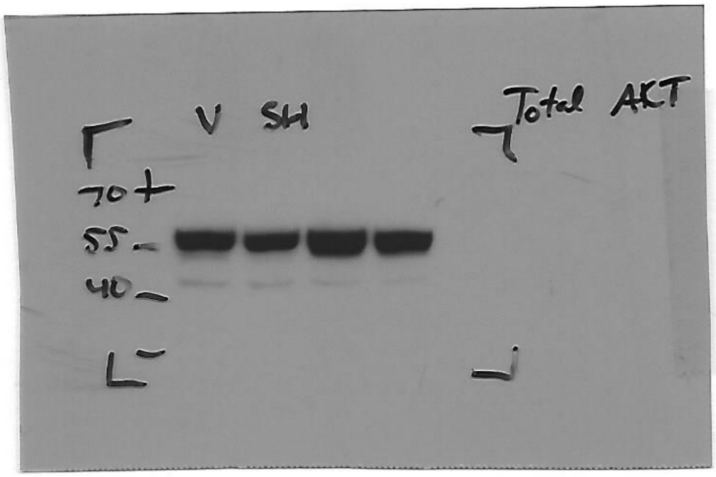

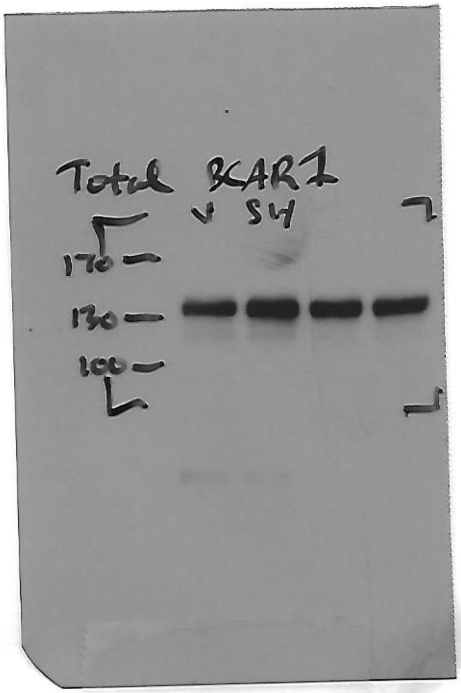

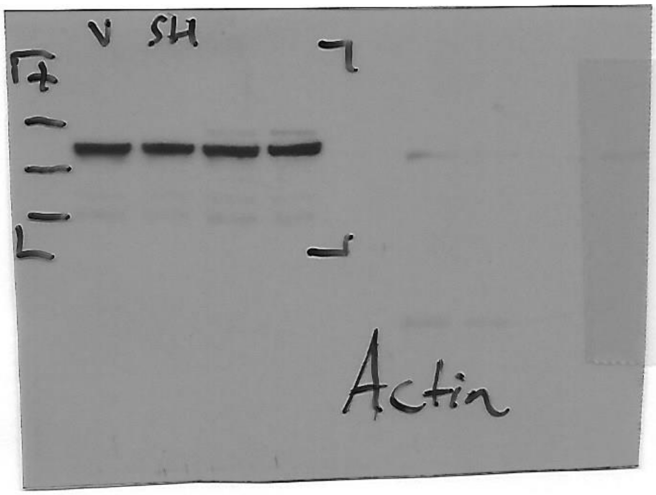

Figure 2-B

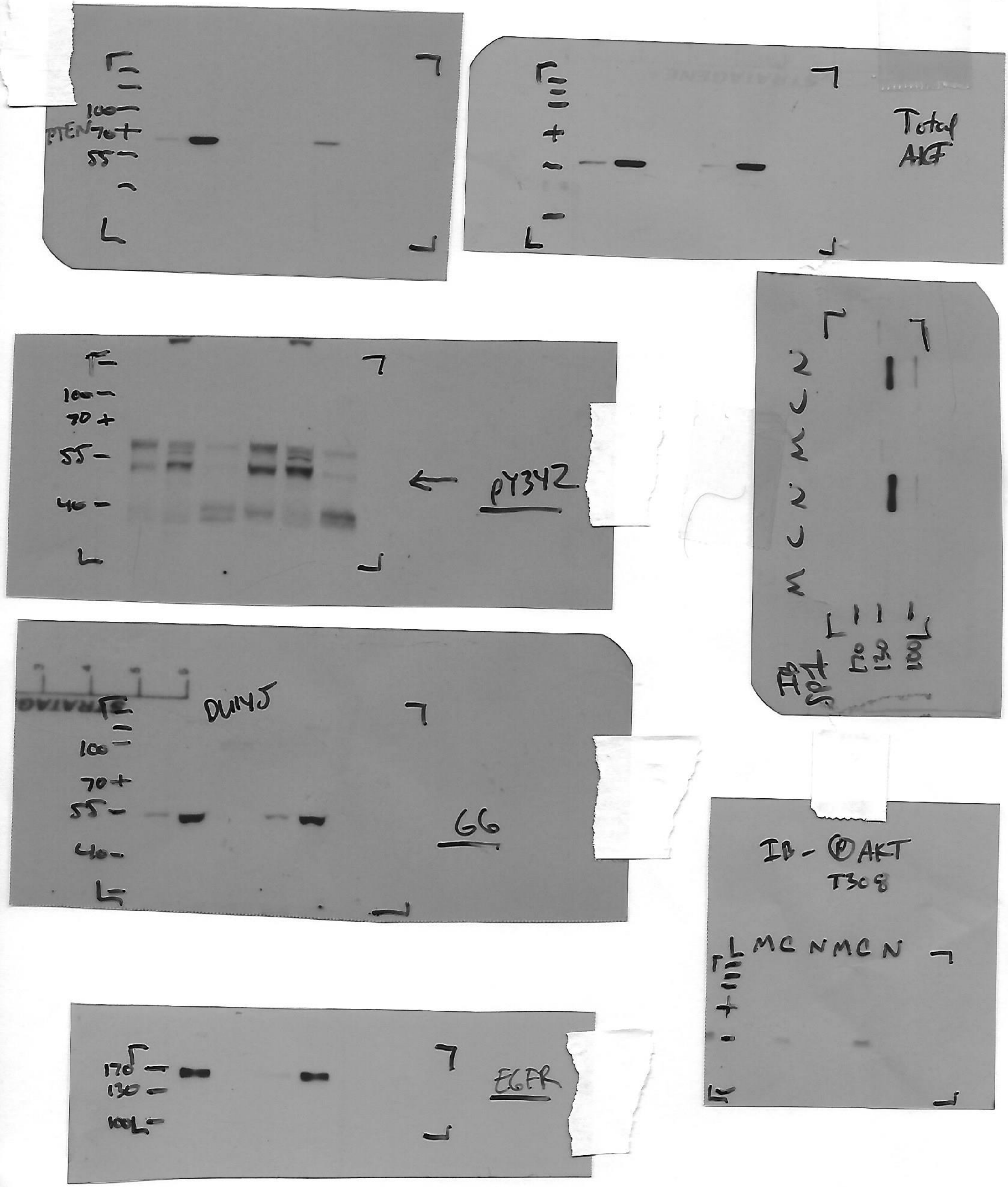

PTEN
100
70
55
Total AKT
100
70
55
40
pY342
DU145
100
70
55
40
66
IB - ⓟ AKT
T308
L M C N M C N
170
130
100
EGFR

Figure 2-E

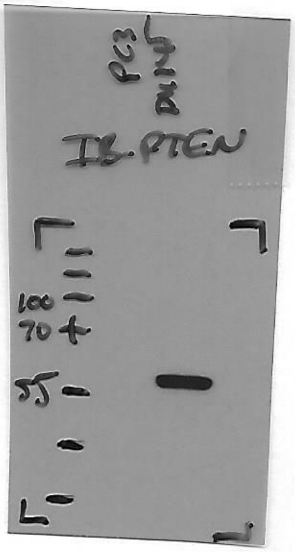
PC3
DU145
IB-PTEN
100
70
35

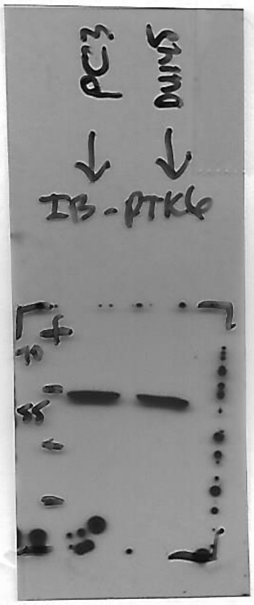
PC3
DU145
IB-PTK6
70
55

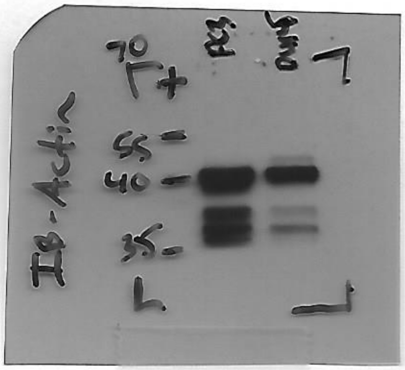
70
PC3
DU145
IB-Actin
55
40
35

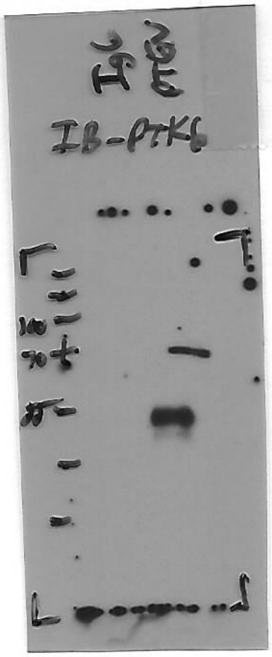
IgG
PTEN
IB-PTK6
100
70
55

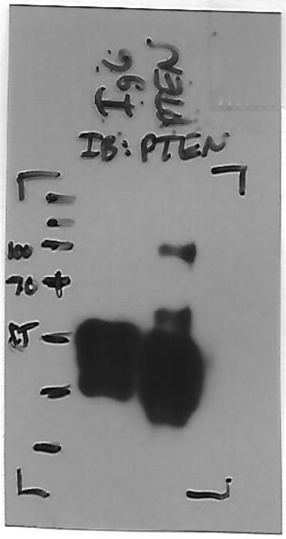
IgG
PTEN
IB: PTEN
100
70
55

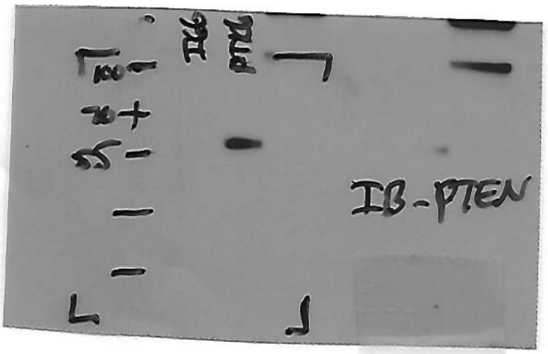
100
70
55
IgG
PTK6
IB-PTEN

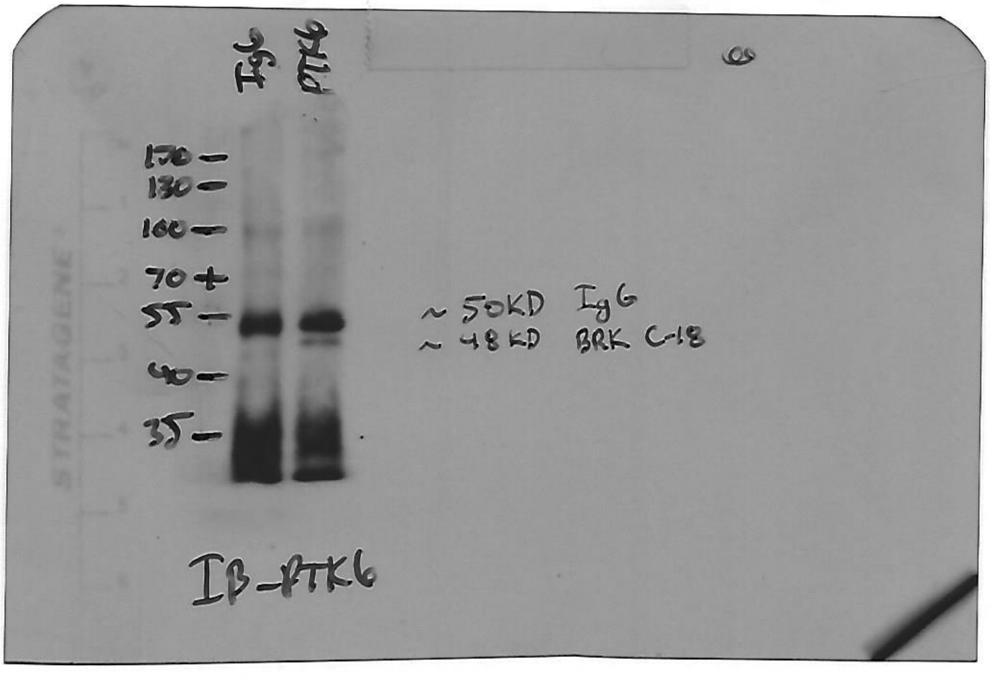
IgG
PTK6
170
130
100
70
55
40
35
~ 50KD IgG
~ 48KD BRK C-18
IB-PTK6

GST - Pull down

10-8-15

Figure 3

A

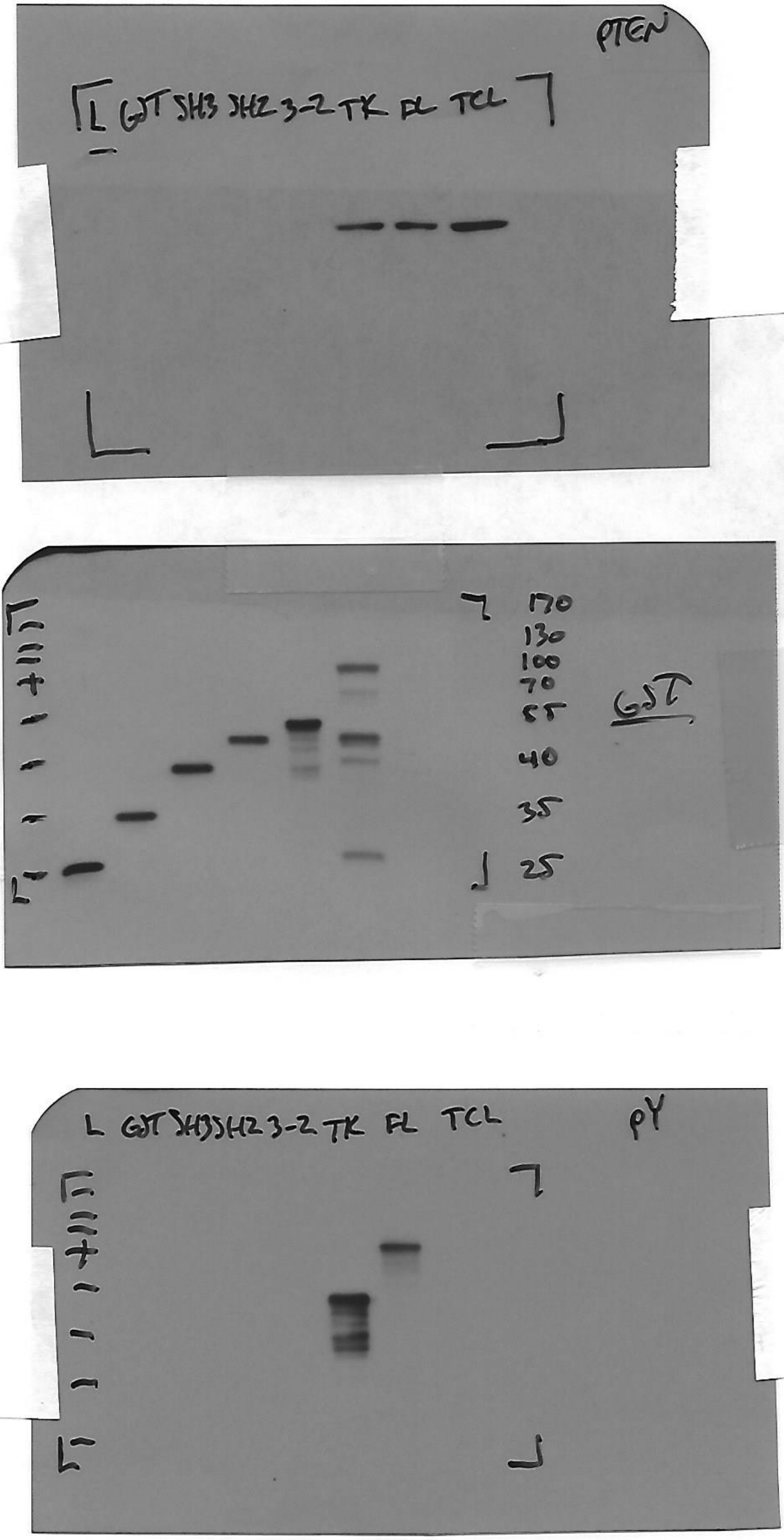

Figure 3-C Trial 1- Phosphatase Assay

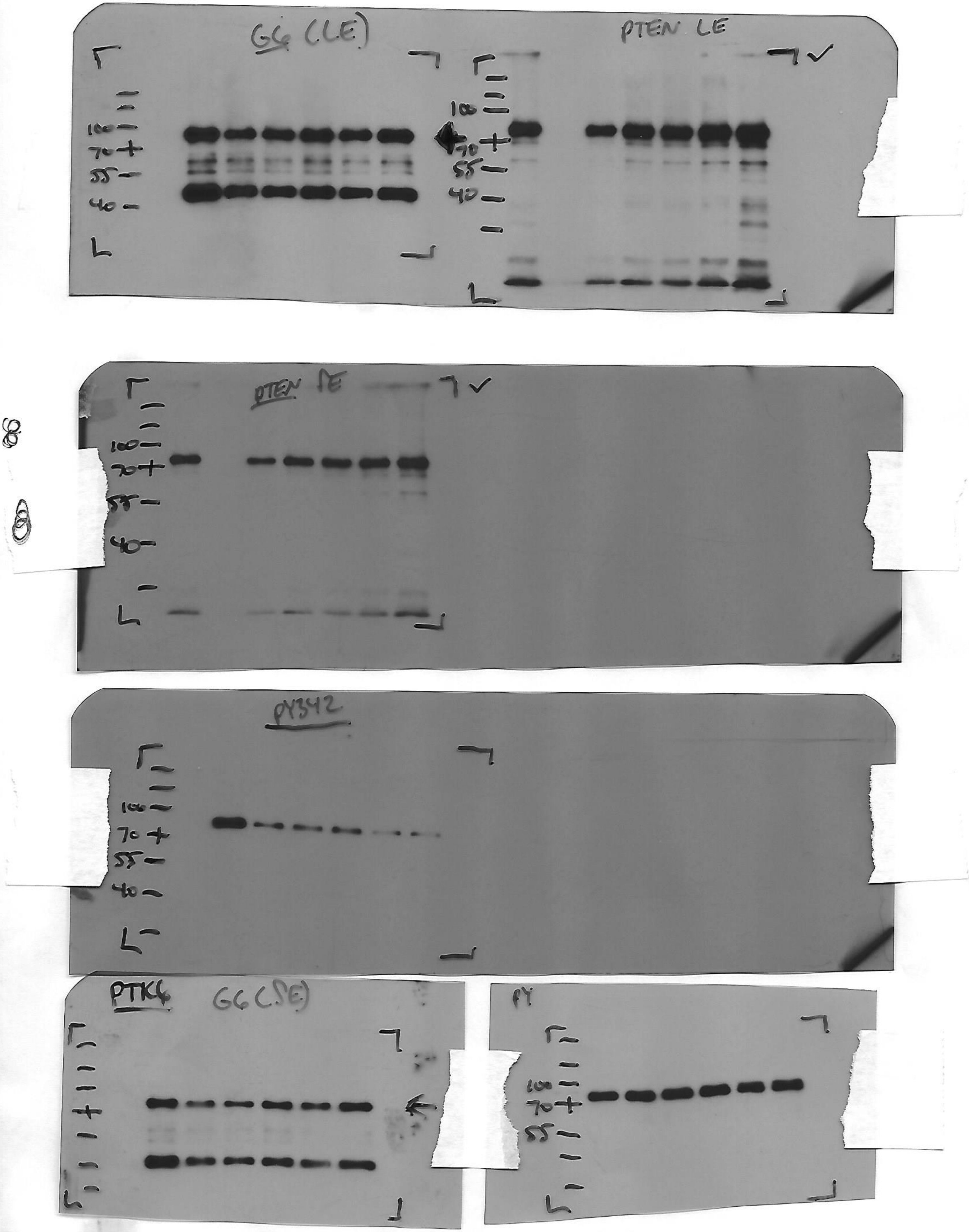

Figure 3
E

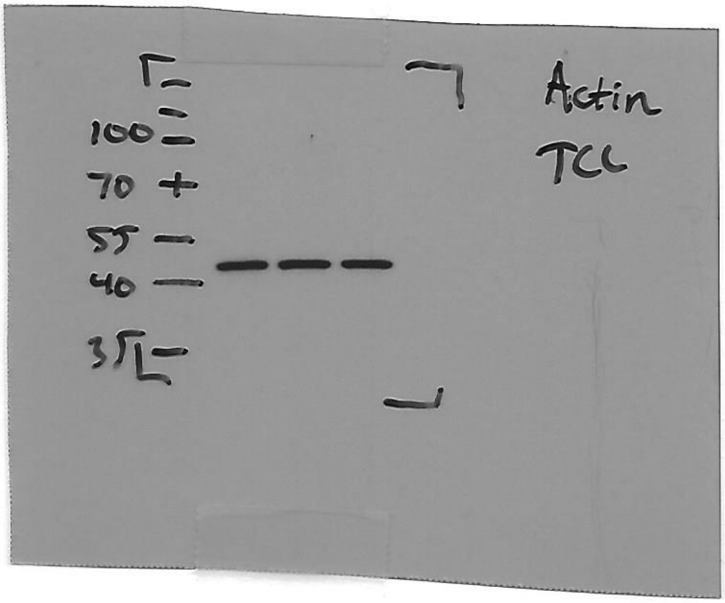

Actin
TCL
100
70
55
40
35

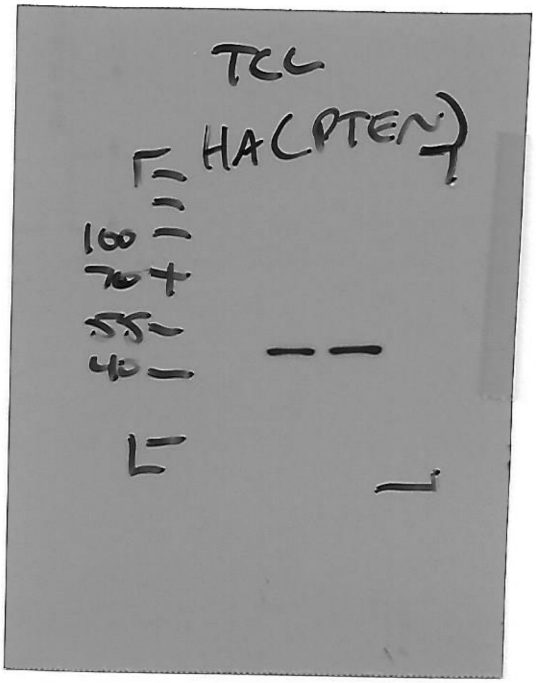

TCL
HA(PTEN)
100
70
55
40

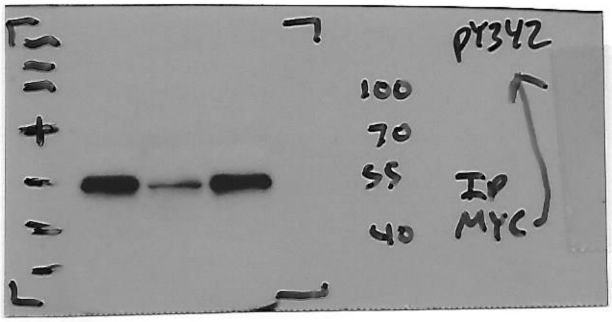

pY342
100
70
55
40
IP
MYC

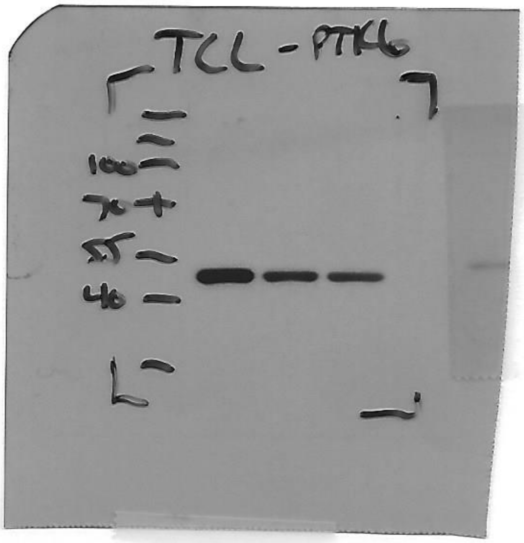

TCL - PTK6
100
70
55
40

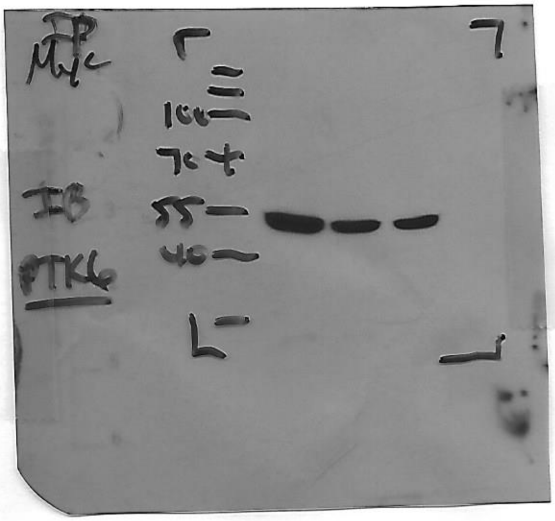

IP
Myc
IB
PTK6
100
70
55
40

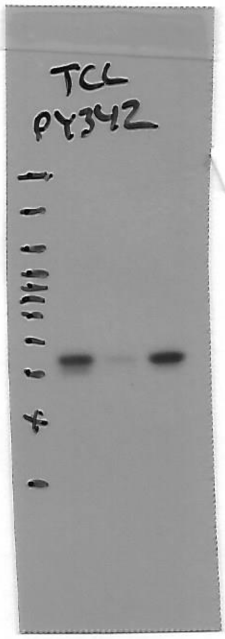

TCL
pY342

Figure 4 B

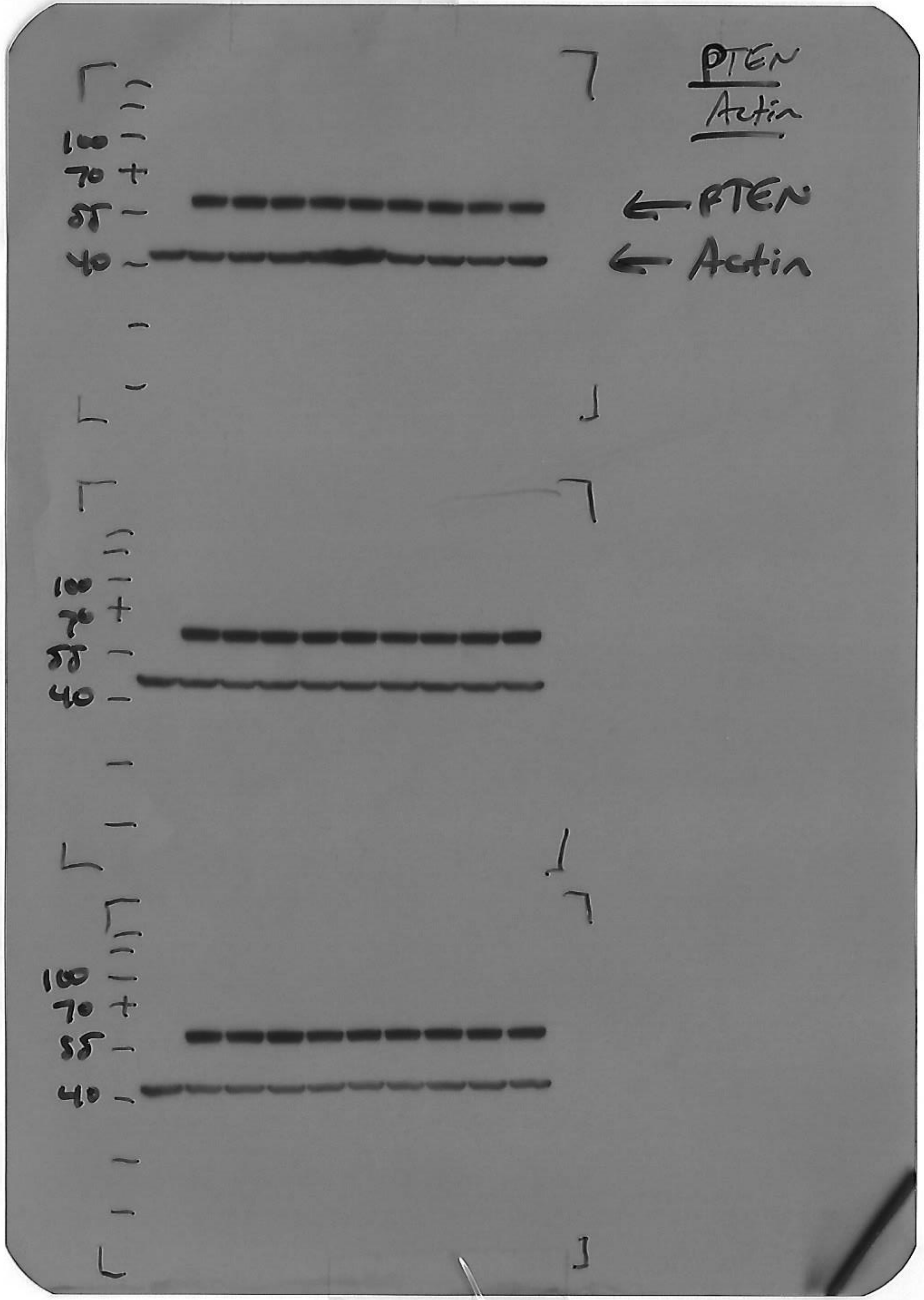
PTEN
Actin
← PTEN
← Actin
100
70
55
40
100
70
55
40
100
70
55
40

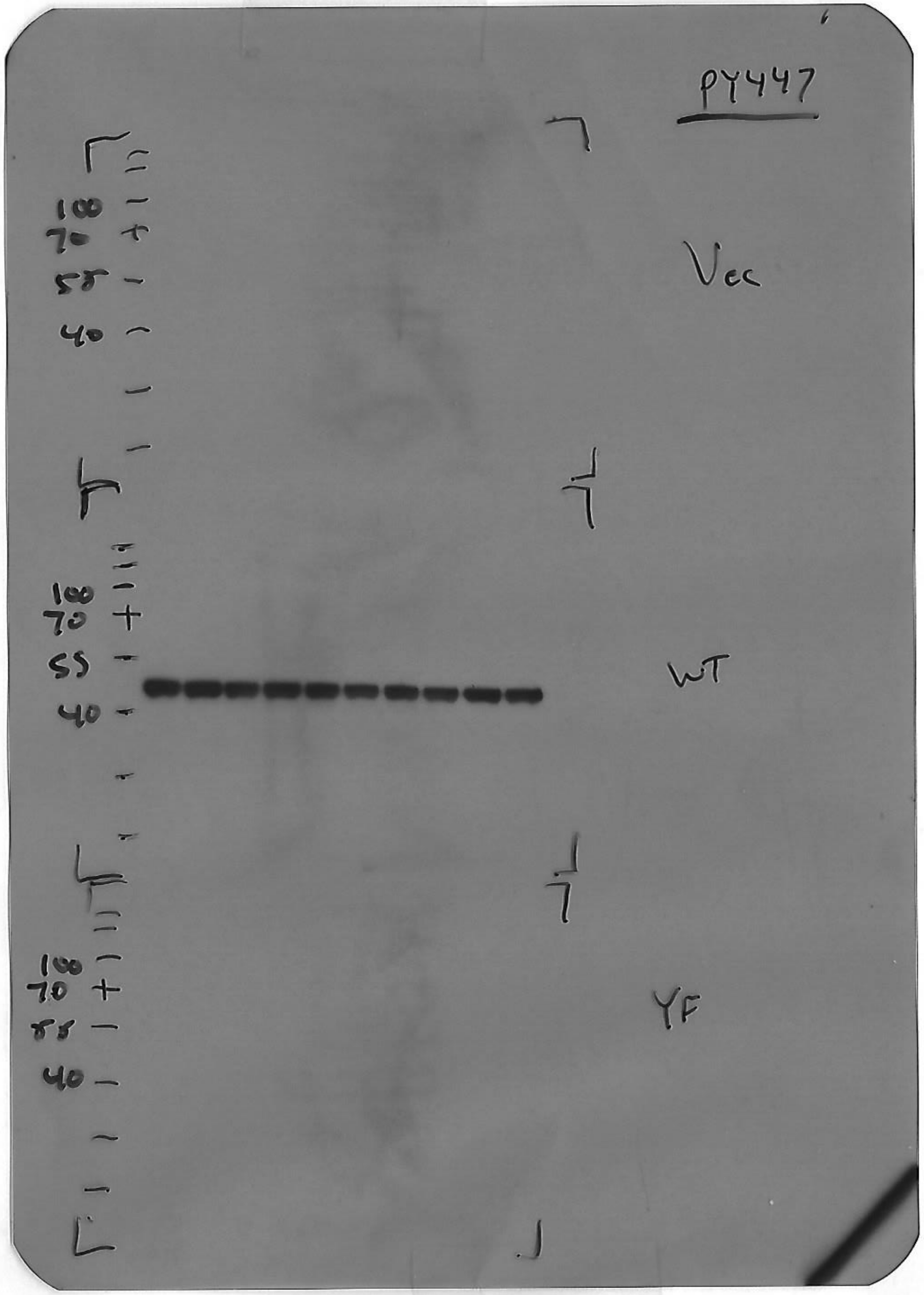
PY447
Vec
WT
YF
100
70
55
40
100
70
55
40
100
70
55
40

Figure 4
B

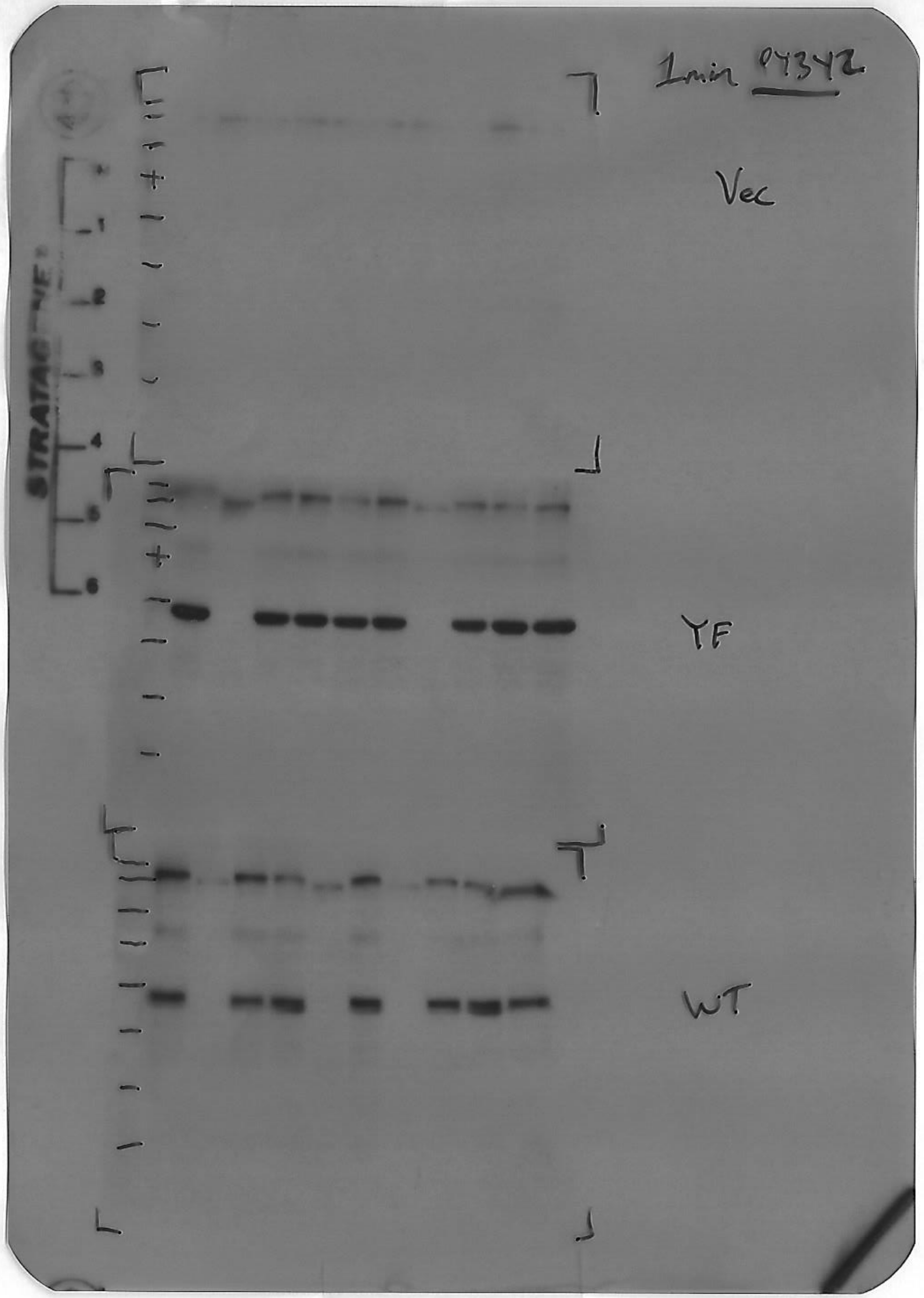

1min PY342
Vec
YF
WT
STRATAGENE

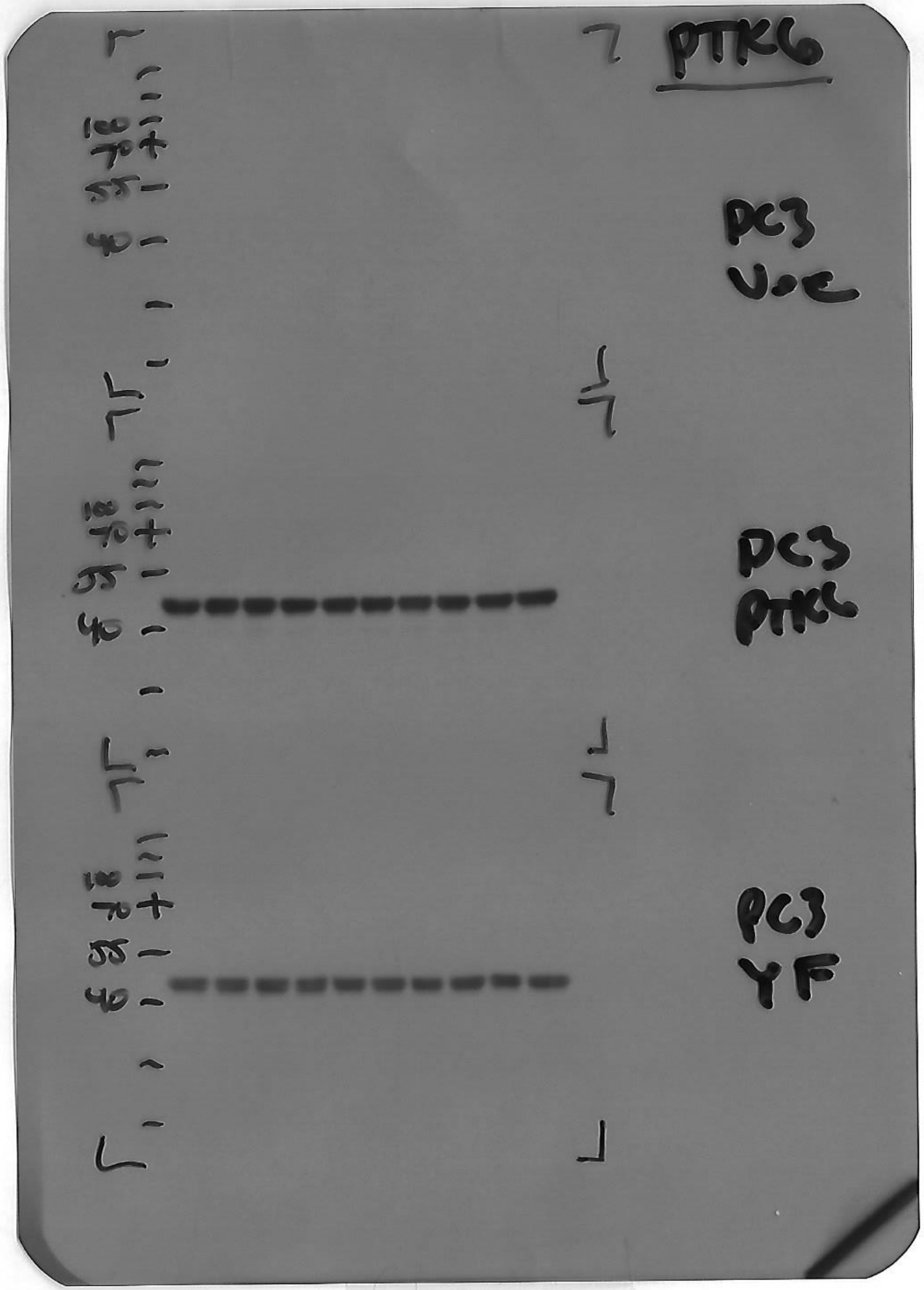

PTK6
PC3
Vec
PC3
PTK6
PC3
YF
100
70
55
40

Figure 4

C.

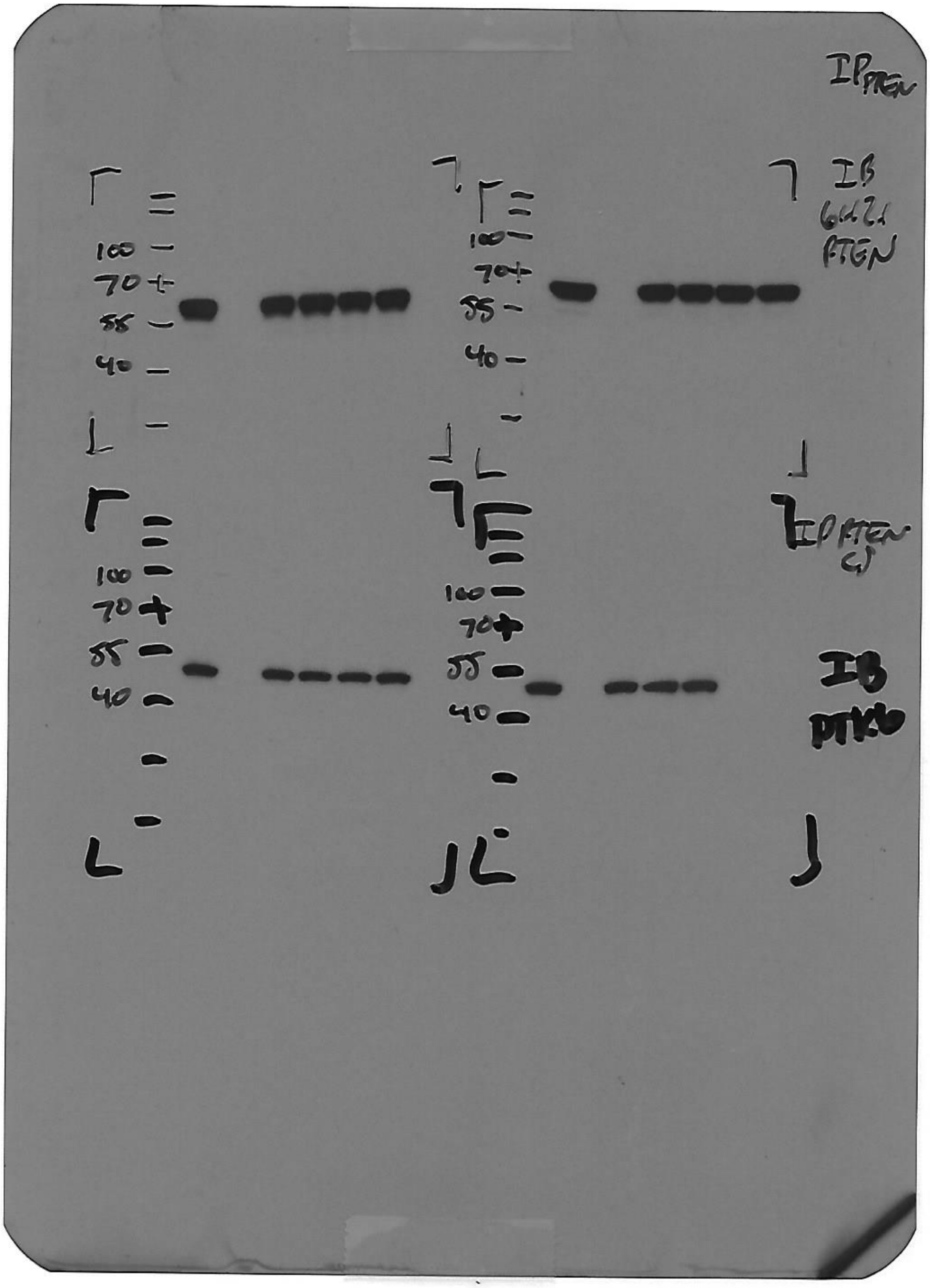

Figure 4E

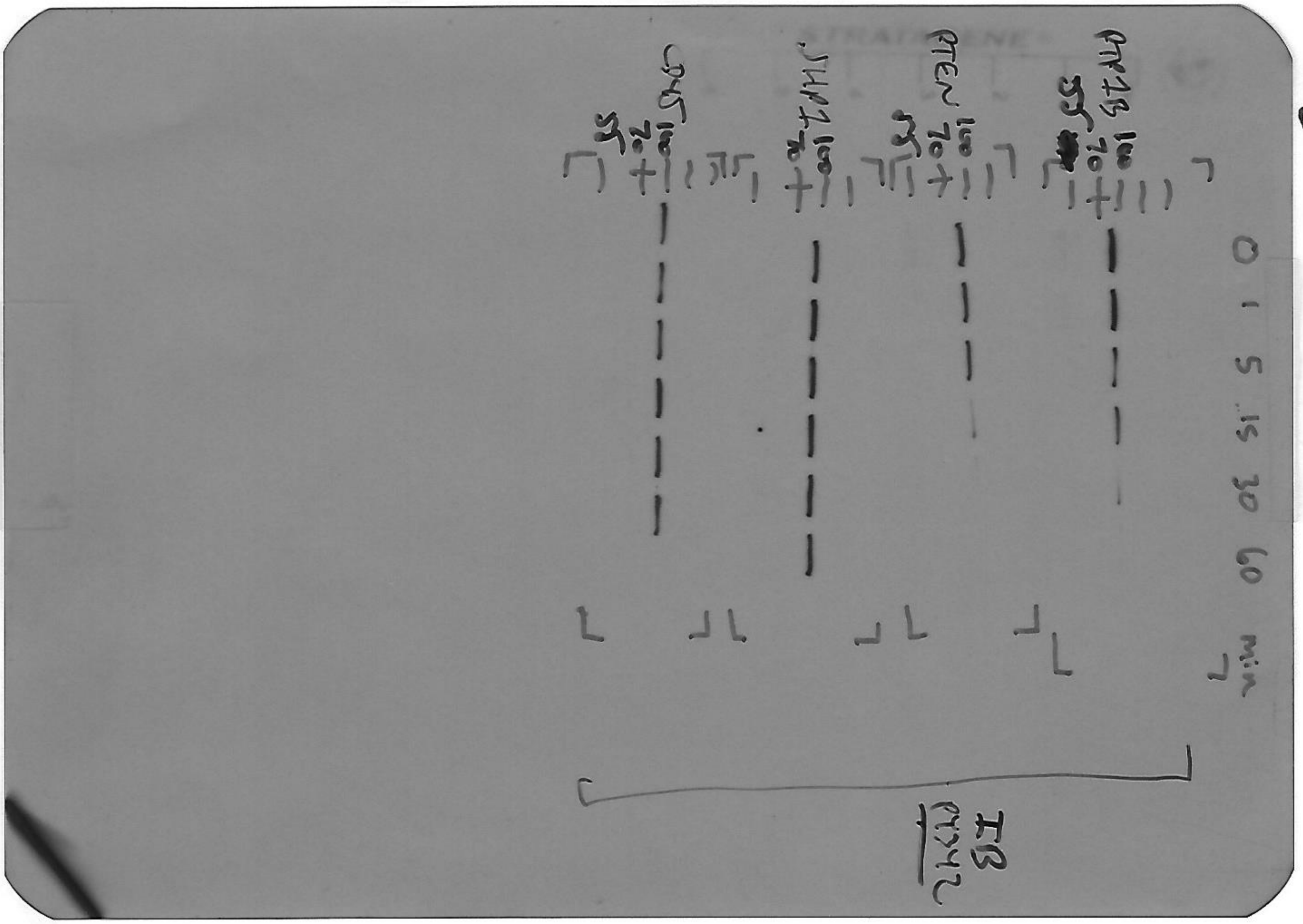
CD45
SHP1
PTEN
PTP1B
0 1 5 15 30 60 min
IB

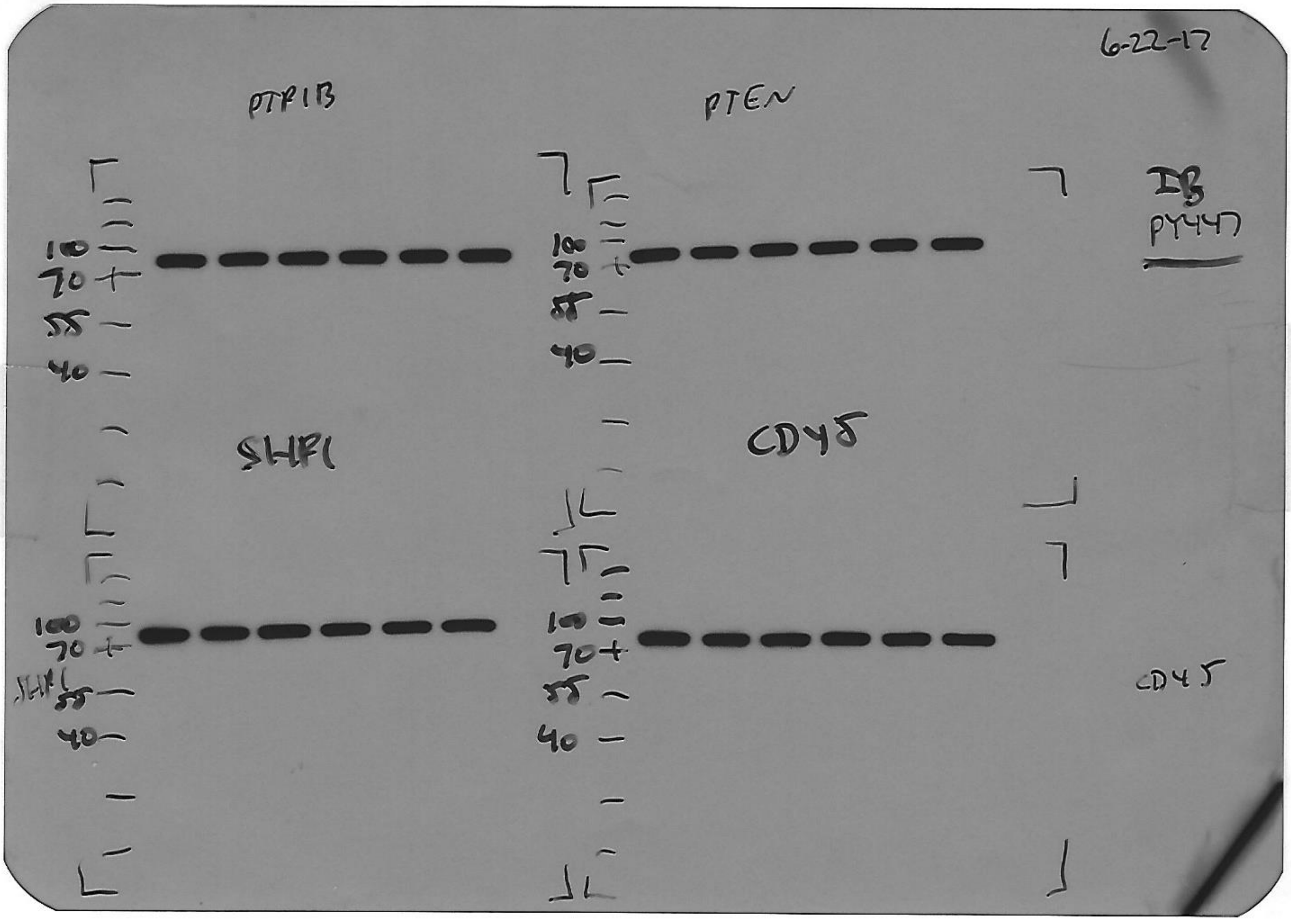
6-22-17
PTP1B
PTEN
IB
PY447
SHP1
CD45
100
70
55
40
CD45

Figure 4 E

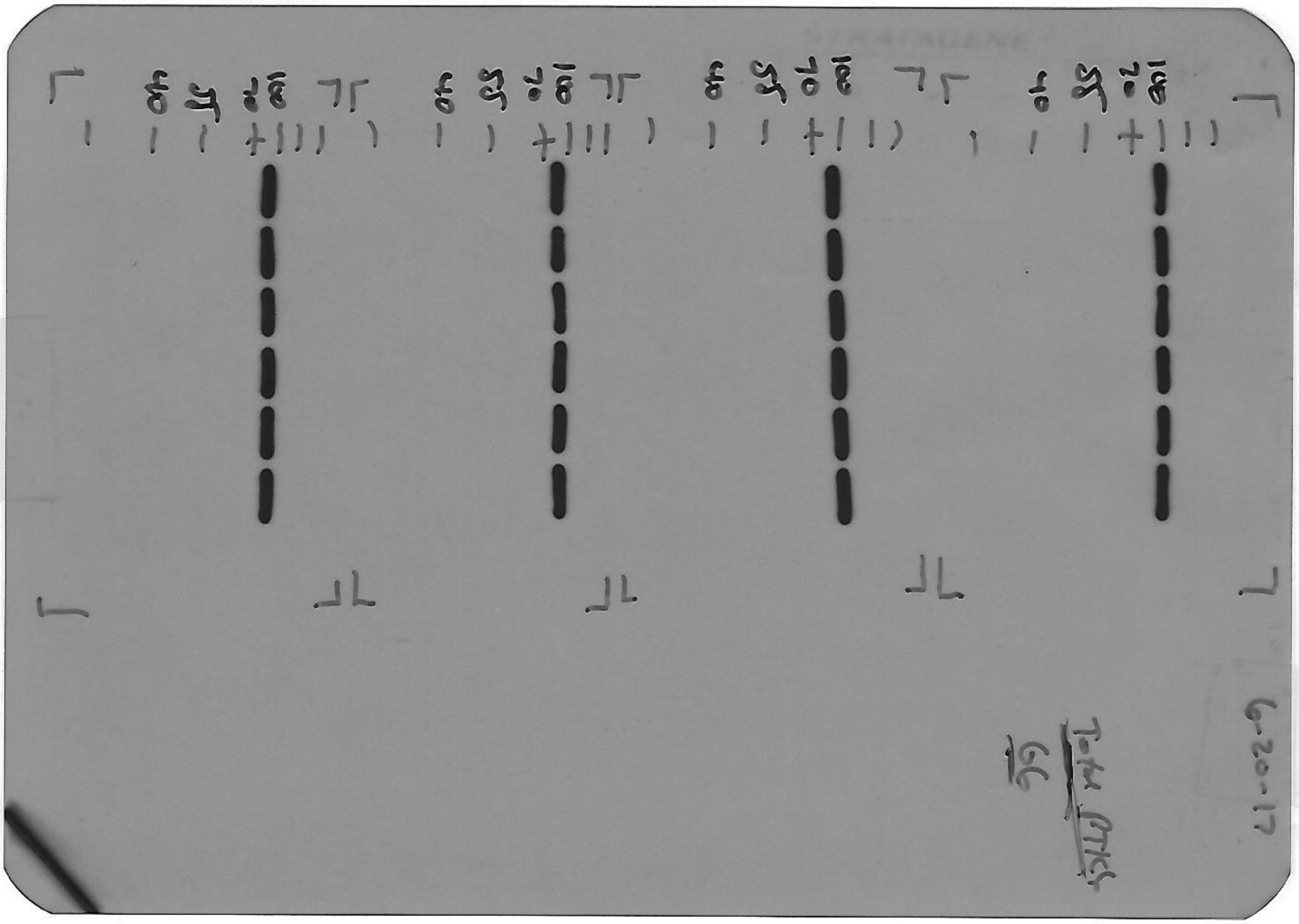

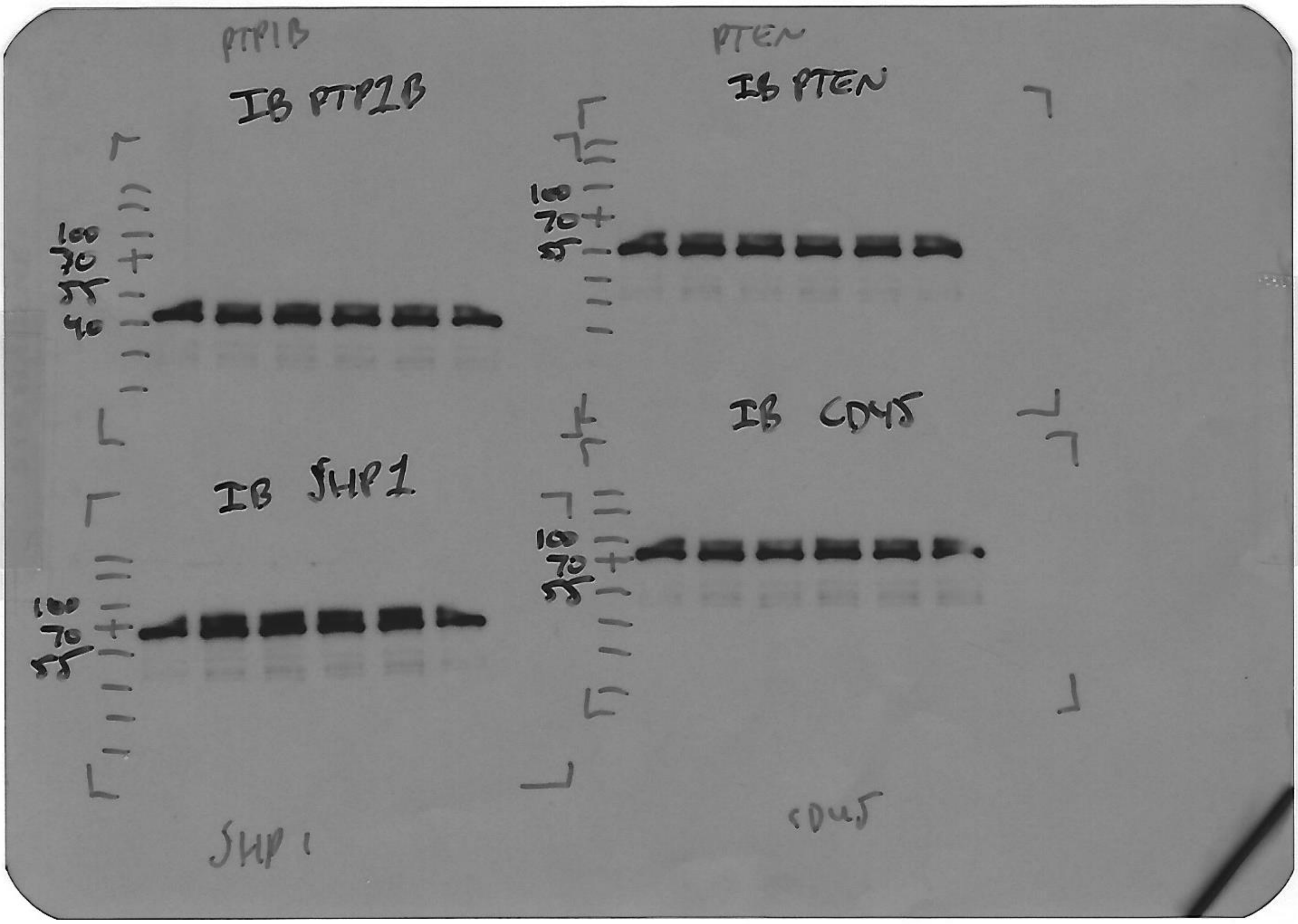

Supplement: Supplementary file 1 — Supplementary Information [file 41467_2017_1574_MOESM1_ESM.pdf]
